# Imaging cancer metabolism using magnetic resonance

**DOI:** 10.1038/s44303-023-00004-0

**Published:** 2024-01-11

**Authors:** Kevin M. Brindle

**Affiliations:** Cancer Research UK Cambridge Institute, University of Cambridge, Li Ka Shing Centre, Robinson Way, Cambridge, CB2 0RE UK

**Keywords:** Biochemistry, Cancer, Oncology

## Abstract

The challenge in clinical oncology is to select the most appropriate treatment for an individual patient. Transcriptome and metabolite profiling have revealed that tumours can display metabolic subtypes with different therapeutic vulnerabilities^1–4^. Metabolic imaging has the potential to distinguish these subtypes and therefore those treatment(s) that should be most effective. Moreover, since changes in tumour metabolism can occur early during treatment, metabolic imaging can also be used subsequently to detect early evidence of treatment response. In this Perspective I briefly review and discuss the relative advantages and disadvantages of magnetic resonance imaging of tumour metabolism using hyperpolarized ^13^C- and ^2^H-labelled substrates.

## Metabolic imaging with Positron Emission Tomography (PET)

PET measurements of 2-deoxy-2-[^18^F]fluoro-D-glucose ([^18^F]FDG) uptake have been widely used clinically for tumour staging, prognosis and treatment monitoring, where it can be more sensitive at detecting treatment response than MRI- and CT-based measurements of tumour size^5^. [^18^F]FDG is taken up on the glucose transporters and trapped in the cell by phosphorylation, catalysed by the first enzyme in the glycolytic pathway, hexokinase. Quantitative measurements of [^18^F]FDG uptake are commonly assumed to provide a measure of glycolytic activity. However, while [^18^F]FDG is a good substrate for the facilitated glucose transporters (GLUTs) it is not a substrate for the sodium-dependent glucose transporters (SGLTs). As a consequence, there can be a disconnect between [^18^F]FDG uptake and glucose utilization. For example, early-stage lung adenocarcinomas in a murine tumour model expressed high levels of SGLT2 activity, whereas GLUT expression was increased in more advanced stages, which can explain why clinically [^18^F]FDG PET has high sensitivity and specificity for detecting the advanced stages of the disease but not the early stages^6^. This problem can be avoided in magnetic resonance studies where the isotope labels (^13^C, ^2^H) have no significant effect on the chemical properties of the labelled molecule.

## Metabolic imaging with magnetic resonance

A limitation of PET is that it only indicates the presence of the radionuclide not what molecule it is in. A key feature of magnetic resonance spectroscopy (MRS) is chemical shift, where the frequency of a resonance is a function of its chemical environment and therefore unlike PET MRS can indicate the molecular origin of the signal.

### ^1^H magnetic resonance spectroscopy

Proton magnetic resonance spectroscopic imaging (MRSI) is an attractive technique for imaging tumour metabolism because of the ubiquity of protons in cellular metabolites and the proton’s high sensitivity to MR detection. The drawbacks include an intense water resonance, which often needs to be suppressed to detect metabolites present in millimolar concentrations, and a narrow chemical shift range that can result in crowded and poorly resolved spectra.

^1^H MRS has been used to detect and grade tumours and monitor their response to treatment, notably in breast, prostate and brain tumours (reviewed in refs. ^7–9^). In brain an increase in the total choline signal (tCho) and a decrease in the concentration of N-acetylaspartate (NAA), a neuronal cell marker, is generally considered as a diagnostic marker of a brain tumour while an increase in the signal at 1.3 ppm from lipid droplets is associated with a higher grade and poorer survival. The tCho/NAA signal ratio was shown to distinguish recurrent glioma from the necrosis resulting from radiation treatment, however its ability to discriminate between the two is inferior to that of perfusion imaging^10^. Mutations in isocitrate dehydrogenases (IDH) 1 and 2 in grade 2 and grade 3 gliomas can be detected very specifically through the resultant accumulation of 2-hydroxyglutarate^11^, which has prognostic value and allows detection of response to IDH inhibitors. The major diagnostic challenge in prostate cancer is to distinguish those tumours that require treatment. Decreased signals from citrate and creatine and increased signals from tCho have been used to detect the presence of cancer and the tCho/creatine signal ratio has been shown to correlate with tumour grade. In breast cancer the tCho signal can be used to distinguish benign from malignant lesions and the water-to-fat signal ratio is higher in invasive ductal carcinoma compared to benign lesions. As in brain tumours a decrease in tCho signal can provide an early indication of response to treatment. Although used widely, and despite numerous clinical studies, ^1^H MRS has yet to become a routine tool in clinical oncology. Although included in a structured reporting system for multiparametric MRI of the prostate it was dropped from a later version because the technique was thought to be difficult to apply in a routine clinical setting. In breast, while it can improve the specificity of a cancer diagnosis, it is limited by relatively long signal acquisition times and chest wall motion. ^1^H MRSI in the brain is more promising where the lack of motion and high filling factor of the detector coil makes it easier to acquire high quality MRSI data.

### ^13^C magnetic resonance spectroscopy

^1^H spectra provide a metabolite profile of a tumour at a point in time. If the metabolism of the tumour is in a steady-state then the technique provides no information on metabolic fluxes, which can often be more informative. Measurement of metabolic flux in this situation requires the introduction of an isotope label. An early example, which illustrates the advantage of measuring metabolic flux, was in stroke, where localized ^1^H NMR spectra showed a high lactate concentration in the infarct. However, this could not distinguish between residual lactate that had pooled in a metabolically inactive region from lactate produced from ongoing glycolytic activity. Infusing the patient with [1-^13^C]glucose resulted in splitting of the lactate resonance due to ^1^H-^13^C spin-spin coupling, demonstrating that the high steady-state lactate concentration was the result of ongoing production from glucose. ^1^H detection of the ^13^C label enhanced the sensitivity of ^13^C detection while also giving the fractional labelling, which at ~50% of the blood glucose fractional labelling demonstrated that most of the observed lactate was derived from glucose metabolised in the glycolytic pathway^12^.

### Hyperpolarized ^13^C magnetic resonance spectroscopy

While direct ^13^C detection, and indirect detection via ^1^H, have been used in clinical studies^13,14^ a general lack of sensitivity has inhibited their widespread adoption. Imaging metabolism using ^13^C labelled substrates, however, underwent a revolution with the introduction of dissolution dynamic nuclear polarization (DNP), which can enhance the sensitivity of ^13^C label detection by > 10,000-fold. DNP involves mixing the ^13^C-labelled molecule with a stable radical and cooling to ~1.2 K in a strong magnetic field. At this temperature the electron spins on the radical become completely polarized and irradiation at the electron spin resonance frequency transfers polarization from the electron spins to the nuclear spins resulting in a substantial increase in nuclear spin polarization. The sample can then be warmed rapidly to room temperature with substantial retention of this nuclear spin polarization^15^. One of the first molecules to be polarized was [1-^13^C]pyruvate. When injected intravenously there was now sufficient signal to image its location in the body and exchange of the hyperpolarized ^13^C label with endogenous lactate, in the reaction catalysed by lactate dehydrogenase (LDH). The main drawback is the relatively short lifetime of the polarization, which in [1-^13^C]pyruvate is ~20 –30 s, which means that imaging needs to be completed within 2–3 min of dissolution. Despite this limitation the technique translated to the clinic in 2013^16^ and there have now been clinical studies in prostate, breast, brain, renal and pancreatic cancer^17,18^. Early preclinical studies showed that lactate labelling from hyperpolarized [1-^13^C]pyruvate could be used to assess tumour grade^19^ and early treatment response^20^ and this has also been demonstrated in clinical studies, including detection of an early response to immune checkpoint therapy in a prostate cancer patient^21^. Lactate labelling is dependent on pyruvate delivery to the tumour, its uptake on the monocarboxylate transporters (MCTs) and label exchange in the reaction catalysed by LDH. In breast tumours there appeared to be a correlation with tumour hypoxia as lactate labelling correlated with the expression of HIF1α and consequent increased expression of MCT1^22^. The technique could also potentially be used to identify tumour metabolic subtypes and therefore to stratify patients for treatment. Lactate labelling was high in some orthotopically implanted patient-derived glioblastoma xenografts whereas in others it was no greater than in normal appearing brain tissue^23^. The same observations were made in glioblastoma patients^24^. RNA sequencing of the tumour model with high levels of lactate labelling showed this to be a mesenchymal subtype^25^ and treatment studies with chemoradiation showed this to be more radioresistant than an oxidative neural progenitor like glioblastoma model with low levels of lactate labelling^23^.

The short half-life of the nuclear spin polarization means that the technique is restricted to studying relatively rapid metabolic reactions and yet despite this limitation many more substrates than might have been anticipated have been used successfully in vivo^26^. These have included H^13^CO_3_^−^ ^27^ and a ^13^C-labelled pyruvate derivative^28^, that can be used to measure tissue extracellular pH. Hyperpolarized [1,4-^13^C_2_]fumarate has been developed as an agent for imaging necrotic cell death^29^ and will translate to the clinic in the coming year. Most viable cells take up fumarate relatively slowly but in necrotic cells, where there is a loss of plasma membrane integrity, fumarate can rapidly gain access to the enzyme fumarase, either intracellularly or in the extracellular space. Fumarase has a high specific activity, requiring only the presence of water to hydrate fumarate to produce malate and therefore increased production of hyperpolarized [1,4-^13^C_2_]malate is a sensitive indicator of cell death.

There are currently around 50 clinical DNP-based hyperpolarizers world-wide and more than 200 human subjects have participated in clinical trials^18^, which have demonstrated the potential of the technique. An important issue now is ease of use, which may yet determine whether the technique is adopted more widely in the clinic. DNP hyperpolarizers require on-site pharmacy facilities and significant in-house expertise to operate. Remote centralised production, where the material is polarized and then shipped at low temperature to the scanner site, may remove the requirement for in-house expertise, requiring only an on-site dissolution device. This may also enable better quality control and uniformity of polarization and reduce costs. Remote production became possible by replacing the stable radical used in the DNP process with a radical obtained by UV-treatment of the pyruvate. This can be annihilated by transiently raising the temperature above the ~ 1.2 K used for polarization and allows storage and transport of the frozen polarized material^30^. Continued presence of the radical would relax the polarization. An alternative approach to polarization is to transfer spin order in parahydrogen to the ^13^C nucleus, in a process that takes a few minutes and which can be accomplished at room temperature. [1-^13^C]pyruvate can be hyperpolarized by using the propargylic ester. Following reduction with parahydrogen the polarization in the resulting allyl ester is then transferred to the ^13^C nucleus before the ester is rapidly hydrolysed to produce hyperpolarized [1-^13^C]pyruvate^31^. A commercial preclinical device is available^32^ with a clinical device planned. A limiting factor in their development has been effective removal of the toxic rhodium catalyst used in the hydrogenation reaction. In principle the devices could be push button with reagents provided in pre-packaged modules that can be plugged into the machine, thus removing the need for significant on-site expertise. The only limitation is that the levels of polarization that can be achieved are less than those that can be achieved using DNP, although this may still be sufficient for many clinical studies.

### ^2^H magnetic resonance spectroscopy

A new approach to metabolic imaging with MRI arrived with the publication of two landmark papers in 2017^33^ and 2018^34^ which showed that ^2^H MRSI could be used to image the metabolism of [6,6-^2^H_2_]glucose in brain, in the latter study in patients with glioblastoma. The fast T_1_ relaxation of the ^2^H nucleus in glucose and its metabolites means that it’s relative insensitivity to MR detection can be compensated by rapid signal acquisition and signal averaging in the absence of significant signal saturation. The natural abundance ^2^H signal in water prior to administration of a labelled substrate provides an inbuilt concentration standard which allows signal intensities to be converted into absolute concentrations^35^. Oral administration of labelled glucose in a glioblastoma patient resulted in detectable labelling of lactate, providing a measure of glycolytic flux, and labelling of glutamate/glutamine (Glx), which provided a surrogate measure of TCA cycle flux. Glycolytic flux was higher and TCA cycle flux lower in the tumour when compared to the surrounding normal appearing brain parenchyma. In a subcutaneous tumour model, a series of rapidly acquired images following intravenous administration of [6,6-^2^H_2_]glucose showed signals from glucose and labelled lactate. Fitting of these signals to a kinetic model of the glycolytic pathway produced a quantitative map of glycolytic flux in the tumour, in mM/min, and showed that this flux decreased dramatically within 24 h of treatment with chemotherapy^36^. Other deuterium-labelled substrates have also been investigated, including acetate, pyruvate, choline and fumarate^37^ and it seems likely that others will be tested in the future. Deuterium labelled substrates can report on metabolic fluxes that are too slow to be detectable using hyperpolarized ^13^C-labelled substrates because of the short polarization lifetime. For example, although flux of hyperpolarized ^13^C label has been detected from [U-^2^H, U-^13^C]glucose through all 10 steps of the glycolytic pathway to lactate in a tumour model this experiment is unlikely to work in clinical studies because of the short polarization lifetime in [U-^2^H, U-^13^C]glucose^38^. However, even for ^2^H-labelled substrates the rate of utilization may still need to be relatively high so that their labelled metabolites can accumulate to high enough concentrations to be detectable.

**Fig. 1:**
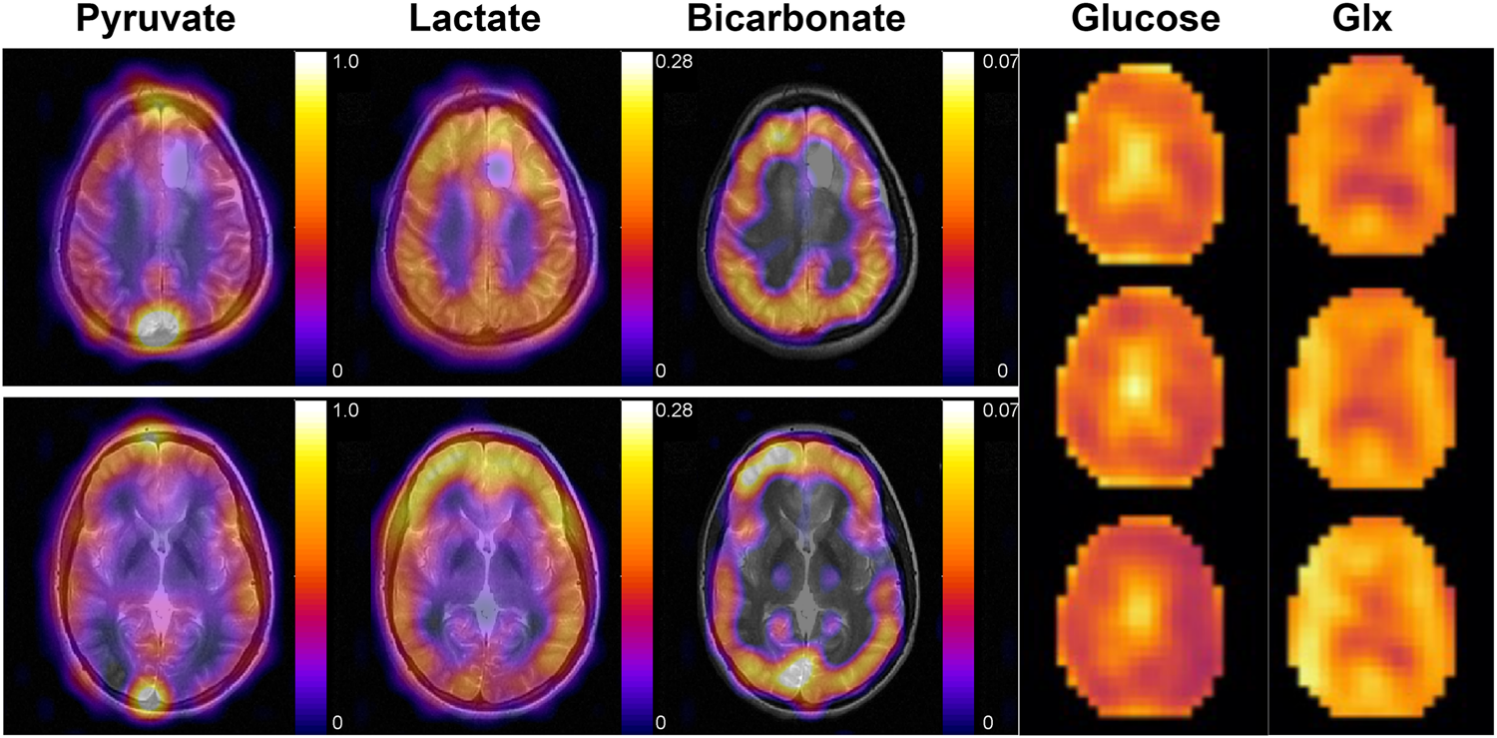
^13^C MR images, acquired at 3 T, of hyperpolarized ^13^C-labelled pyruvate, lactate and bicarbonate (overlaid on a T_2_-weighted ^1^H image) and ^2^H MR images, acquired at 9.4 T, of ^2^H-labelled glucose and Glx in the brains of healthy volunteers. The ^13^C images are the sum of 20 images acquired over 60 s following the intravenous injection of 0.43 mL/kg of ~250 mM hyperpolarized [1-^13^C]pyruvate. The nominal image resolution was 1.5 × 1.5 × 2 cm^3^. The ^2^H images show three 10 min images, acquired between 91.5 and 121.5 min after oral administration of 0.75 g/kg body weight of [6,6’-^2^H_2_]glucose. The nominal image resolution was 1.5 × 1.54 × 1.29 cm^3^. Bicarbonate and Glx labelling are higher in the grey matter, reflecting increased TCA cycle activity in grey matter as compared to white matter. The images were taken, with permission, from^49,50^.

Deuterium metabolic imaging (DMI) with [6,6-^2^H_2_]glucose could be used analogously to hyperpolarized [1-^13^C]pyruvate for imaging tumour subtype, assessing grade and detecting response to treatment. [2,3-^2^H_2_]fumarate has already been shown to be potentially superior to hyperpolarized [1,4-^13^C_2_]fumarate in detecting tumour cell death post-treatment^39^ and has the advantage that it can be administered orally^40^. Although the signal-to-noise ratio in the hyperpolarized ^13^C experiment was higher the contrast developed in the ^2^H experiment was greater as the build-up of labelled malate could be detected over a much longer period of time, giving a higher malate/fumarate ratio and therefore greater image contrast^41^. The relative advantages and disadvantages of imaging metabolism with hyperpolarized ^13^C- and ^2^H-labelled substrates are summarized in Table 1. Imaging of brain metabolism using hyperpolarized [1-^13^C]pyruvate and [6,6’-2H2]glucose is illustrated in Fig. 1.

**Table 1:** A comparison of the relative advantages and disadvantages of imaging metabolism with hyperpolarized ^13^C- and ^2^H-labelled substrates.

^13^C	^2^H
Complex equipment required for hyperpolarization.	Material can be taken off the shelf
Administered intravenously	Can be administered orally
Can be implemented at clinical magnetic field strengths (e.g., 3 T)	Although can be used clinically at 3 T^51^, the narrow frequency range and broad resonances means that it works better at higher field strengths, where spectral resolution improves linearly with field and sensitivity improves with the magnetic field to a power of + 1.65^52^.
Short polarization lifetimes make it logistically more challenging to administer a hyperpolarized ^13^C-labelled substrate in the clinic.	The material is stable and therefore quality control is not required immediately prior to administration.
Polarization is short-lived and therefore the technique can only interrogate relatively rapid metabolic reactions.	The ^2^H label is stable and therefore in principle can be used to interrogate slower metabolic reactions.
The high sensitivity of detection means that relatively low concentrations can be used, avoiding potential toxicity problems.	The low sensitivity of detection requires relatively high concentrations of the labelled material to be administered, which clinically may restrict use to substrates with a very low toxicity profile.
Detection of the ^13^C label requires the scanner to have X nucleus capability.	The ^2^H label can be detected indirectly via the loss of ^1^H signal intensity^53^.

## Evaluation of hyperpolarized ^13^C and ^2^H MRS(I) in comparison with existing metabolic imaging methods

The rapid metabolic changes that often accompany tumour treatment means that pyruvate and glucose can be used to detect very early evidence of treatment response while imaging with fumarate can be used to determine whether this translates into subsequent tumour cell death. Since [^18^F]FDG-PET is already used in the clinic to detect treatment response and diffusion-weighted ^1^H imaging, for example, has been used clinically to detect cell death from the resulting loss of tumour cellularity it seems appropriate to ask what advantages ^13^C MRSI with hyperpolarized [1-^13^C]pyruvate and [1,4-^13^C_2_]fumarate and ^2^H MRSI with [6,6-^2^H_2_]glucose and [2,3-^2^H_2_]fumarate might have over these existing techniques. This is not to suggest that these ^13^C and ^2^H MRSI experiments will replace existing PET and ^1^H MR(S)I techniques, for example it is difficult to see how they could compete with the sensitivity of PET for detecting the presence of disease, particularly with the introduction of “Total Body PET”, with its attendant increases in whole body coverage and detection sensitivity^42^. However, if these hyperpolarized ^13^C and ^2^H MR techniques are to be adopted in the clinic then they need to demonstrate some clear and compelling advantages over the techniques already in use.

In an ovarian cancer model treated with a tyrosine kinase inhibitor there was an early increase in lactate labelling with no change [^18^F]FDG uptake^43^ and in colorectal xenografts, in which cell death was induced with a TRAIL agonist, there was a decrease in lactate labelling but no change in [^18^F]FDG uptake despite a decrease in glycolytic flux and lactate production measured using ^13^C-labelled glucose (non-hyperpolarized)^44^. In a study of ER+ breast cancer models treated with a PI3K inhibitor lactate labelling was shown to be decreased due to decreased expression of the transcription factor FOXM1, which in these ER+ tumours drives expression of LDH. However, there was no change in expression of c-Myc or HIF-1α and no change in expression of the GLUTs or hexokinase and therefore no change in [^18^F]FDG uptake^45^. In the case of cell death detection, ^2^H MRSI of [2,3-^2^H_2_]fumarate metabolism detected cell death in orthotopically implanted glioblastoma models treated with chemoradiation earlier than detection using diffusion-weighted or contrast agent-enhanced ^1^H MRI^41^. In summary, there are already preclinical examples which have shown that under some circumstances these hyperpolarized ^13^C- and ^2^H-based MR metabolic imaging methods may detect an early response to treatment where none is detected using imaging methods that are already employed in the clinic.

## Outlook

For ^13^C and ^2^H MRSI to be used more widely in the clinic these techniques will need to address outstanding clinical questions that cannot adequately be addressed using existing clinical methods. The barriers that they face are similar to those that were faced by proton MRSI. Cost is less important (the costs for a hyperpolarized [1-^13^C]pyruvate or [6,6-^2^H_2_]glucose exam are similar^37^) when one considers the costs of modern cancer treatments, however ease of use and data interpretability certainly are. Widespread adoption will require large scale, well controlled multi-site clinical trials that demonstrate utility in specific applications. A problem with proton MRSI was data interpretation and the same will apply to ^13^C and ^2^H MRSI. The application of radiomics and artificial intelligence may help here by providing an automated, objective and quantitative interpretation of the data without having to rely on the more subjective assessment of a resident expert^46^. I began my career using ^1^H NMR to study ^1^H/^2^H exchange in lactate in erythrocyte suspensions ^47,48^. In the last 40 years it has become evident that this and other MRS techniques can be used clinically to interrogate the biology of various tissues in the clinic. The challenge remains to turn these into methods that are used widely and routinely to answer important clinical questions.

## Data Availability

No datasets were generated or analysed during the current study.
